# Tissue Factor-Targeted “O_2_-Evolving” Nanoparticles for Photodynamic Therapy in Malignant Lymphoma

**DOI:** 10.3389/fonc.2020.524712

**Published:** 2020-11-10

**Authors:** Ziying Li, Yanxue Yin, Weiwei Jin, Bo Zhang, Han Yan, Heng Mei, Huafang Wang, Tao Guo, Wei Shi, Yu Hu

**Affiliations:** ^1^ Institute of Hematology, Union Hospital, Tongji Medical College, Huazhong University of Science and Technology, Wuhan, China; ^2^ Targeted Biotherapy Key Laboratory of Ministry of Education, Wuhan, China; ^3^ Department of Cardiovascular, Optical Valley School District, Hubei Province Academy of Traditional Chinese Medicine, Wuhan, China; ^4^ Department of Systems Biology, National Cancer Institute Comprehensive Cancer Center, Beckman Research Institute, City of Hope, Monrovia, CA, United States

**Keywords:** catalase, photodynamic therapy, tissue factor, nanoparticles, EGFP-EGF1protein

## Abstract

Vascular-targeted PDT (vPDT) has produced promising results in the treatment of many cancers, including drug-resistant ones, but little is known about its efficacy in lymphoma. Unfortunately, the lack of a specific therapeutic target and a hypoxic microenvironment for lymphoma jeopardizes the efficacy of vPDT severely. In this study, we designed a lymphoma tissue factor-targeted “O_2_-evolving” strategy combining PDT with catalase and HMME-encapsulated, EGFP-EGF1-modified PEG-PLGA nanoparticles (CENPs) to boost PDT efficiency; this combination takes advantage of the low oxygen tension of lymphoma. In our results, CENPs accumulated effectively in the vascular lymphoma *in vivo* and *in vitro*, and this accumulation increased further with PDT treatment. Per positron emission tomography imaging, combining CENPs with PDT inhibited lymphoma glucose metabolism significantly. The expression of hypoxia-inducible factor (HIF)-1α in the entrapped catalase groups reduced markedly. These data show that the combined administration of PDT and CENPs can prompt tissue factor-cascade-targeted and self-supply of oxygen and that it has a good therapeutic effect on malignant lymphoma.

## Introduction

Despite the development of new therapies, patients with partial non-Hodgkin’s lymphoma (NHL) are still treatment-resistant; the hypoxia attributed to malformed vasculature and rapid tumor growth may be the real problem (1, 2). So, it is imperative that more effective and safer treatment strategies for NHL be found.

Vascular-targeted photodynamic therapy (vPDT) is employed as an antitumor regime by taking advantage of tumor vascular-localizing photosensitizers (PS), appropriate wavelength light, and spot irradiation of tumor areas, which change oxygen molecules into reactive oxygen species (ROS) and damages vessels, thereby starving tumors into regression (3, 4). vPDT has been used to treat many tumors in the preclinic and clinic (5–7), but it has never been used to treat NHL, except cutaneous T-cell lymphoma (8).

The most important step in the killing ability of PDT is the tumor gathering of PS and local ROS production (9). Unlike normal tissues, tumor vascular endothelia highly express the tissue factor (TF), transforming it into a specific tumor vascular target (10, 11). In the process of PDT, TF could abundantly generate at vascular sites due to the damage of ROS to endothelial cells, so more PS could be recruited through the positive feedback effect. In previous studies, we prepared a TF-targeted drug delivery system successfully: EGFP-EGF1-NPs-loaded HMME. The system, composed of a factor VII-derived EGFP-EGF1 fusion protein and PEG-PLGA nanoparticle-loaded hematoporphyrin monomethyl ether (HMME), combined with PDT to exhibit efficient TF-cascade-targeting ability *in vitro* and *in vivo* (12).

However, phototoxic ROS produced by PDT depends on O_2_, and solid tumor tissue hypoxia (usually ≤ 15 mmHg) restricts PDT photochemical reactions; O_2_ consumption and the breakdown of blood vessels aggravate hypoxia further, hampering the killing effect of PDT (13, 14). To combat hypoxia during the PDT process, some researchers have, in the past, incorporated catalase into the aqueous core of nanoparticles for reaction with hydrogen peroxide (H_2_O_2_) in the tumor microenvironment to guarantee continuous O_2_ supply and, thus, exert a stronger killing effect (15, 16).

In the present study, the advantages of ROS triggering TF cascade targeted effect and catalase improving hypoxia microenvironment ability were utilized to enhance vPDT treatment on malignant lymphoma. We first constructed a TF-cascade-targeted “O_2_-evolving” system—catalase and HMME-encapsulated, EGFP-EGF1-modified PEG-PLGA nanoparticles (CENPs)—to target malignant lymphoma vessels. The self-amplified targeting property and O_2_ production capacity of CENPs coupled PDT were investigated both *in vitro* and *in vivo*. Finally, the pharmacodynamics experiment was evaluated and compared with other groups.

## Material and Methods

### Materials

EGFP-EGF1 was acquired from Amoytop Biotech (Xiamen, China). Maleimide-PEG-OH (MW 3500 Da) was obtained from Jenkem (China). Maleimide-PEG-PLGA (MW 10000 Da, Mn 96494, IP 1.04, copolymer ratio 50:50) and Methoxy-PEG-PLGA (MW 96000 Da, Mn 86266, IP 1.11, copolymer ratio 50:50) were purchased from the Institute of medical instruments of Shandong (China). Hematoporphyrin monomethyl ether (HMME) was procured from Dibo Chemical Technology Co., Ltd. (Shanghai, China). Ammonium molybdate, H_2_O_2_, catalase, Coumarin-6, 40, and 6-Diamidino-2-phenylindole (DAPI) were supplied by Sigma Co. (USA). 2-iminothiolane (Traut’s reagent), BCA Protein Assay Reagent, and [Ru(dpp)_3_]Cl_2_ were obtained from Thermo Fisher Scientific Inc. (USA). Sodium cholate was bought from Shanghai Chemical Reagent Company (China). Ni2t-NTA affinity chromatography and Sephacryl S-100 HR chromatography were acquired from GE Healthcare (USA). Dihydroethidium (DHE) and N-acetylcysteine (NAC) were obtained from Beyotime® Biotechnology Co., Ltd. (Nantong, China). Endothelial Cell Medium (ECM) was purchased from ScienCell (USA). RPMI-1640, fetal bovine serum (FBS), trypsin-EDTA (0.25%), and penicillin/streptomycin were bought from Gibco (CA). DMEM-F12 was supplied by Hyclone (USA). The rabbit anti-rat TF polyclonal antibody was obtained from Abcam (USA). Mouse-derived CD31 polyclonal antibody was acquired from Santa Cruz Biotechnology (USA). HIF-1α Antibody was purchased from Novus Biologicals (USA). Ellman’s reagent was procured from Acros Co. (Bruxelles, Belgium). 18F-fluorodeoxyglucose Positron emission tomography (PET) imaging was performed at the center of the Wuhan Union Hospital, China. All other reagents were commercially available and used without further purification.

### Cell Culture

The human Burkitt lymphoma CA46 cell line was frozen in this laboratory. Cells were maintained in RPMI-1640 supplemented with 10% FBS; the Jeko-1 and Raji cell lines were purchased from the China Center for Type Culture Collection (Wuhan University, China) and cultured in an RPMI-1640 culture medium containing 10% FBS at 37°C in a humidified atmosphere enclosing 5% CO_2_. Human umbilical vein endothelial cells (HUVECs) were purchased from ScienCell (USA) and cultured in a special endothelial cell medium (ECM).

### Animals and Tumor Models

Male non-obese diabetic/severe combined immunodeficient (NOD/SCID) mice (3–4 weeks old) were provided by HFK Bioscience Co. Ltd. (Beijing, China) and maintained under specific pathogen-free conditions. All animal experiments were performed in accordance with protocols evaluated and approved by the ethical committee of Tongji Medical College.

Inoculations with an intradermal injection of 1.2×10^^7^ Jeko-1 cells or 6×10^^6^ Raji cells were performed on the depilated right shoulders of NOD/SCID mice. PDT was conducted when tumors reached a diameter of 7 to 10 mm. A fiber optic bundle of a cold light (540 nm) source (KL 1500 LCD, SCHOTT, Germany), with a 4.5 mm aperture, was suspended 1 mm above the tumors to illuminate them for 15 min at an intensity of 3000 K; illumination started 15 min after the intravenous injection of nanoparticles. Thirty mice were then divided into six groups at random (n=5): normal saline, EGFP-EGF1-conjugated PEG-PLGA nanoparticle-loaded HMME (ENPs), PEG-PLGA nanoparticle-loaded HMME (NPs) plus PDT, catalase and HMME-encapsulated PEG-PLGA nanoparticles (CNPs) plus PDT, ENPs plus PDT, and CENPs plus PDT. Two mg kg^−1^ of nanoparticles were injected into corresponding groups intravenously.

### Preparing Nanoparticles

NPs, ENPs, CNPs, and CENPs were prepared using a double-emulsion solvent-evaporation technique, as described previously, with minor modifications (12, 17). In brief, catalase (2.5 mg) was dissolved in 1 ml of double-distilled water and mixed to prepare the internal water phase (W), and MPEG-PLGA (21.8 mg) and Mal-PEG-PLGA (2.18 mg) were dissolved in 1 ml of dichloromethane to prepare the oil phase (O). To 1 ml of the oil phase was added 50 µl of the internal water phase (internal water phase: oil phase volume ratio = 1:20) in a 50 ml centrifuge tube. The mixture was sonicated (160–200W, 30 s) on ice using a probe sonicator (Scientz Biotechnology Co., Ltd., China) to prepare W/O preliminary emulsion, and then 2 ml of 1% sodium chlorate aqueous solution was added to the sonicated mixture (160–200W, 1s) on ice for 20 times to obtain W/O/W double emulsion. After evaporating dichloromethane with a ZX-98 rotary evaporator (Shanghai Institute of Organic Chemistry, China) at 37°C, the obtained nanoparticles were concentrated by centrifugation at 14,500 rpm for 45 min using a TJ-25 centrifuge (Beckman Coulter, USA). The supernatant was discarded subsequently, and the nanoparticles were re-suspended in 200–400 µl of PBS (0.01 M, pH =7.0). Thiolated EGFP-EGF1 proteins were added to the CNPs, and the remaining steps were performed in a manner consistent with the preparation of ENPs to obtain CENPs. HMME-loaded, coumarin-6, or Dir-labelled nanoparticles were prepared using the same procedure, except that 4 mg of HMME, 30 mg of coumarin-6, or 200 mg of Dir was added additionally to dichloromethane that contained copolymers before primary emulsification.

### Characterizing Nanoparticles

The morphology of nanoparticles was observed using an H-600 transmission electron microscope (TEM, Hitachi, Japan). A Zeta Potential/Particle Sizer NICOMP™380 ZLS (PSS Nicomp Particle Size System, USA) was used to measure the mean diameter and polydispersity index (PDI) of nanoparticles by employing dynamic light scattering with a He-Ne laser at 632.8 nm. The concentrations of coumarin-6 and HMME (wave length 395 nm) were determined using an established standard curve line based on their absorbance spectra in acetonitrile. The Dir concentrations were measured by UV spectrophotometry at a wavelength of 758 nm. Drug-loading capacity (DLC) was determined based on the ratio of the final drug weight to the overall weight of the nanoparticles. Bubble formation was observed by adding H_2_O_2_ directly to CNPs, and catalase activity was determined using the Goth method (18).

### Uptake of Nanoparticles by HUVECs

HUVECs were incubated with coumarin-6-CENPs (100 ng/mL of coumarin-6) for 1 h, 3 h, 6 h, and 10 h to observe the time trend, fixed with 4% paraformaldehyde after the nanoparticles were washed off, and then the slips were rinsed and incubated with DAPI for examination with the fluorescence microscope (Ex/Em: 466/504 nm) (Leica, DMI4000B, Germany).

For the examination of intracellular uptake, HUVECs were co-incubated with coumarin-6-labeled NPs, CNPs, ENPs, and CENPs for 6 h and then observed under fluorescence microscopy and flow cytometry (BD, USA).

### *In Vitro* Quantification of TF Expression of HUVECs Post-PDT

HUVECs were incubated with NPs, CNPs, ENPs, and CENPs and then irradiated with 540 nm laser for 30 s. At 1 h post-PDT, cells were incubated with DHE (10 µm) to determine the level of generation of ROS under fluorescence imaging (Ex/Em: 518/605 nm). In the presence of ROS, DHE is oxidized to ethidium, which binds with DNA in the nucleus, producing red fluorescence. For the quantification of TF expression post-PDT, HUVECs were incubated with CENPs (10, 50, 100, 500 ng/mL) for 6 h, lysed with RIPA, and assessed for TF expression using western blotting and real-time PCR.

### *In Vitro* Evaluation of O_2_ Production Induced by Catalase-Encapsulated Nanoparticles in CA46 Cells

This experiment was divided into the following six groups: blank control, NAC, NPs, CNPs, ENPs, and CENPs, and fluorescence microscopy was used to identify the effect of nanoparticles on ROS in CA46 cells. Cells were incubated with nanoparticles for 24 h, followed by another incubation with DHE for 30 min, after which NAC was used to scavenge ROS because of its antioxidant properties; the NAC group was pretreated with NAC for 1 h before DHE was added.

[Ru(dpp)_3_]Cl_2_(Ex/Em: 488/620 nm), an O_2_ sensing probe whose fluorescence can be strongly quenched by O_2_, was used to assess the intracellular generation of O_2_ (19). [Ru(dpp)_3_]Cl_2_ (5 µm) was pre-loaded into CA46 cells by co-incubation for 4 h, incubated for a second time with NPs, CNPs, ENPs, and CENPs for 24 h, and then the cells were subjected to flow cytometry.

### Fluorescence Imaging of Nanoparticles in Tumor-Bearing Mice Post-PDT

Mice were injected with Dir-labelled nanoparticles, and PDT was delivered, as previously reported, for 15 min (20). At various points (3 h, 6 h, 10 h, 24 h) post-PDT, fluorescent scans (Ex/Em: 730/790 nm) were conducted on anesthetized mice using the Maestro 2 *In Vivo* Imaging System (CRI, USA). Mice were euthanized 24 h post-PDT, and tumors were harvested. Each tumor was washed with PBS and then subjected to fluorescence imaging.

### Biodistribution of Nanoparticles and TF Expression in Tumor Vascular Endothelium in Tumor-Bearing Mice Post-PDT

Nanoparticles were labeled using coumarin-6 to observe nanoparticle distribution and TF expression in the tumor vascular endothelium. Animals were killed 6 h post-PDT, and tumors were collected in frozen sections of 5 μm thickness and stained with rabbit polyclonal antibodies specific for tissue factor and mouse polyclonal antibodies specific for CD31. Sections were observed under confocal microscopy (ZEISS, Germany).

### Evaluating O_2_ Production in Tumor-Bearing Mice Post-PDT

Nanoparticles were injected separately, and after PDT, all mice were sacrificed, and the tumors collected were fixed in 4% paraformaldehyde, embedded in paraffin, sectioned at a thickness of 5 μm, and subjected to immunohistochemical staining to analyze the expression of HIF-1α.

### Local Tumor Thrombosis Post-PDT

To observe local tumor thrombosis, two subcutaneous mouse lymphoma models of the Jeko-1 cell line and Raji cell line were established. All mice were sacrificed post-PDT, and tumors were fixed in 4% paraformaldehyde, embedded in paraffin, sectioned at a thickness of 5 μm, and subjected to H&E staining for the observation of thrombosis.

### *In Vivo* Antitumor Study

The Jeko-1 and Raji tumor models were employed for the *in vivo* antitumor study. For the Raji tumor model, small-animal PET (RAYCAN Technology Co., Ltd. [Suzhou]) was used to evaluate the pharmacodynamics of subcutaneous tumors. 18F-Fluorodeoxyglucose PET imaging was performed 24 h before and 24 h after PDT treatment (day 0). PET images were reconstructed using 3D ordered-subsets expectation maximization. The mean radioactivity of the 18F-FDG tumor uptake was calculated with decay correction from the region of interest. The bodyweights and tumor sizes of all mice were monitored every day. On day 14, all the mice received corresponding nanoparticle tail vein injections and PDT treatment again. The mice were sacrificed on day 15, and typical tumors and major organs (heart, liver, spleen, lung, and kidney) were harvested for H&E staining. Tumor apoptosis was also assessed using TUNEL assay according to the instructions that accompanied the product. TUNEL staining was used to study the antitumor effect of PDT on the Jeko-1 tumor model.

### Data Analysis

Statistical analysis was carried out using the GraphPad Prism software. Data are expressed as the means ± standard deviations. The one-way analysis of variance was used to analyze the significance among groups, after which post hoc tests with Bonferroni correction were applied for comparisons between individual groups. P < 0.05 was considered significant.

## Results

### Characterization of Nanoparticles

**Figure 1:**
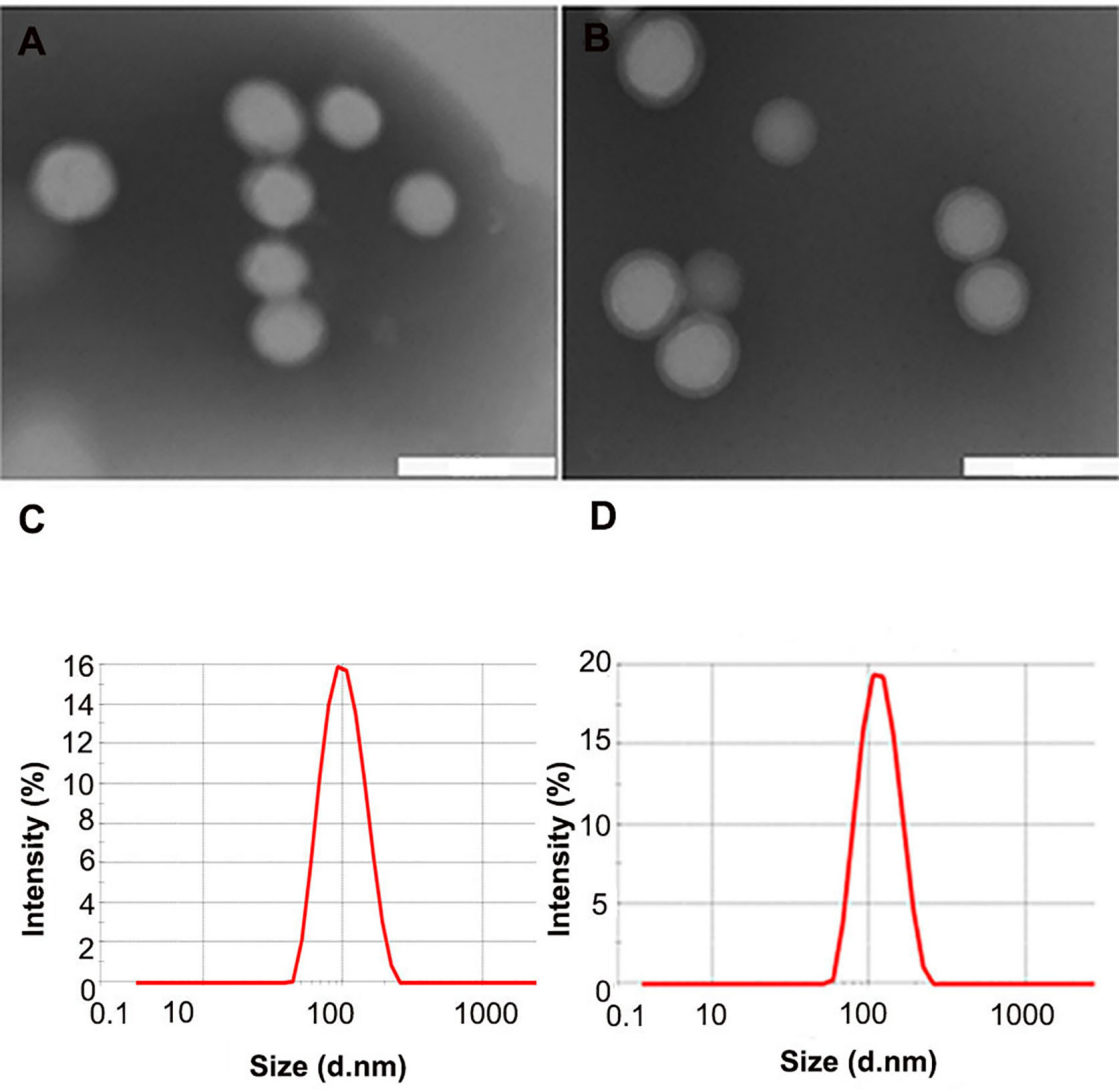
Characteristics of different nanoparticles. **(A, B)** TEM scan of NPs and ENPs; **(C, D)** Size distribution of NPs and ENPs by DLS with He-Ne laser at 632.8 nm. (scale bars 200 nm).

TEM showed that NPs and ENPs were consistent in size and shape and homogeneously distributed (**Figure 1**). As shown in **Table 1**, after EGFP-EGF1 modification and catalase encapsulation, the diameter of NPs increased from 110 nm to 129.3 nm and then to 157.4 nm, all of which are less than 200 nm and would, thus, avoid capture by the liver, indicating a favorable size for drug delivery (21, 22). Furthermore, nanoparticles exhibited excellent stabilities in PBS, RPMI-1640, and 10% FBS without significant aggregation (data not shown). The favorable stability of nanoparticles was demonstrated similar as previously reported (12). Concentrations of HMME, coumarin-6, or Dir in nanoparticles were determined based on their respective absorbance spectra in acetonitrile. The DLC of HMME was 0.5–2%, which far exceeds the dose required.

**Table 1:** Particle size, polydispersity index (PDI), and drug loading capacity (DLC) of nanoparticles. Measured in double-distilled water (n = 3), mean ± SD.

Nanoparticles	Mean size (nm)	PDI	DLC (%)
NPs	110.0±1.70	0.142±0.0318	1.3603±0.0392
CNPs	147.7±0.47	0.105±0.0208	1.0869±0.0287
ENPs	129.3±2.54	0.132±0.0217	0.5571±0.0291
CENPs	157.4±3.07	0.121±0.0249	0.6427±0.0156
Coumarin-6-NPs	123.4±7.04	0.224±0.0272	0.065±0.005
Coumarin-6-CNPs	139.4±2.10	0.192±0.0207	0.062±0.003
Coumarin-6-ENPs	131.1±1.21	0.212±0.0038	0.057±0.007
Coumarin-6-CENPs	146.3±2.95	0.180±0.0207	0.057±0.007
Dir-NPs	108.8±1.55	0.187±0.0061	0.9793±0.0287
Dir-CNPs	159.9±0.69	0.155±0.0239	0.9218±0.0306
Dir-ENPs	138.7±3.08	0.223±0.0102	0.9179±0.0271
Dir-CENPs	162.2±3.65	0.281±0.0114	0.8299±0.0397

Catalase is an enzyme that catalyzes the decomposition of H_2_O_2_ to water and oxygen (23). The CNPs tube bubbled when H_2_O_2_ was added. The immediate formation of abundant O_2_ bubbles revealed the strong catalytic capability of CNPs (**Figure S1**). The catalase activity of CNPs was determined quantitatively using the Goth method and was only 10% of that of free catalase, which may be attributed to the fact that it takes more than 24 h for catalase to break through the surface of nanoparticles before it can be released fully (15). Our findings show that we constructed TF-targeted “O_2_-evolving” nanoparticles successfully.

### Characteristics of the *In Vitro* Uptake of Nanoparticles by HUVECs

**Figure 2:**
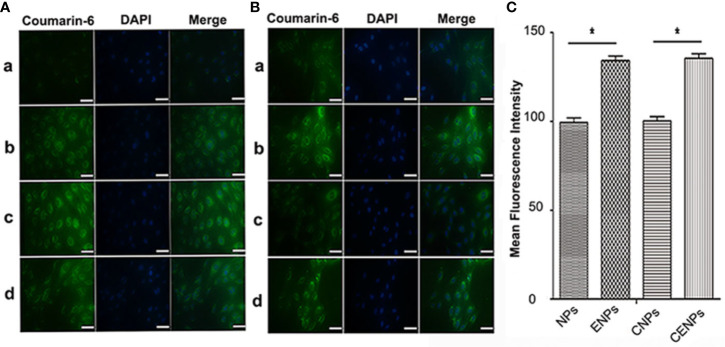
*In vitro* uptake of nanoparticles by HUVECs. **(A)** Time trend of nanoparticle uptake by HUVECs. a. 1 h; b. 3 h; c. 6 h; d. 10 h. **(B)** Uptake of coumarin-6-labelled nanoparticles by HUVECs at 37°C for 6 h under microscopy. a. Coumarin-6-NPs; b. Coumarin-6-ENPs; c. Coumarin-6-CNPs; d. Coumarin-6-CENPs. Green: Coumarin-6-labelled nanoparticles; Blue: Nuclei. (scale bars 50 μm) **(C)** Uptake of coumarin-6-labelled nanoparticles was investigated by flow cytometry. Data are expressed as the mean ± SD (n = 3); *p < 0.05, compared with the control group.

HUVEC was a common cellular that mimicked certain phenotypes of neovascular cells. Coumarin-6 (green) was used as a fluorescence indicator to track nanoparticles taken up by HUVECs. After incubation with coumarin-6-labeled CENPs, the fluorescence of HUVECs increased gradually with incubation time and reached a maximum point after 6 h of incubation, indicating that nanoparticles were taken up efficiently (**Figure 2A**). For that reason, we selected the 6 h mark as the time point to explore the uptake of nanoparticles. As shown in **Figure 2B**, the uptake of coumarin-6-labeled ENPs/CENPs increased significantly, compared with the uptake of NPs/CNPs. Further quantitative analysis by flow cytometry revealed that HUVEC’s uptake of EGFP-EGF1-modified nanoparticles was 1.4 times that of unmodified nanoparticles (**Figure 2C**). These data indicate that EGFP-EGF1 on the surface of nanoparticles could recognize TF specifically and, hence, contribute significantly to the uptake of EGFP-EGF1-modified nanoparticles by HUVECs.

### *In Vitro* Quantification of the TF Expression of HUVECs Post-PDT

To measure ROS levels in HUVECs post-PDT, DHE, one of the most common probes used for superoxide compound anionic fluorescent detection, was utilized. Fluorescence microscopy showed that single irradiation increased ROS production slightly, while the co-incubation of nanoparticles increased the red fluorescence signal significantly. Additionally, remarkably more ROS were detected in the ENPs/CENPs plus PDT groups (**Figure S2**).

**Figure 3:**
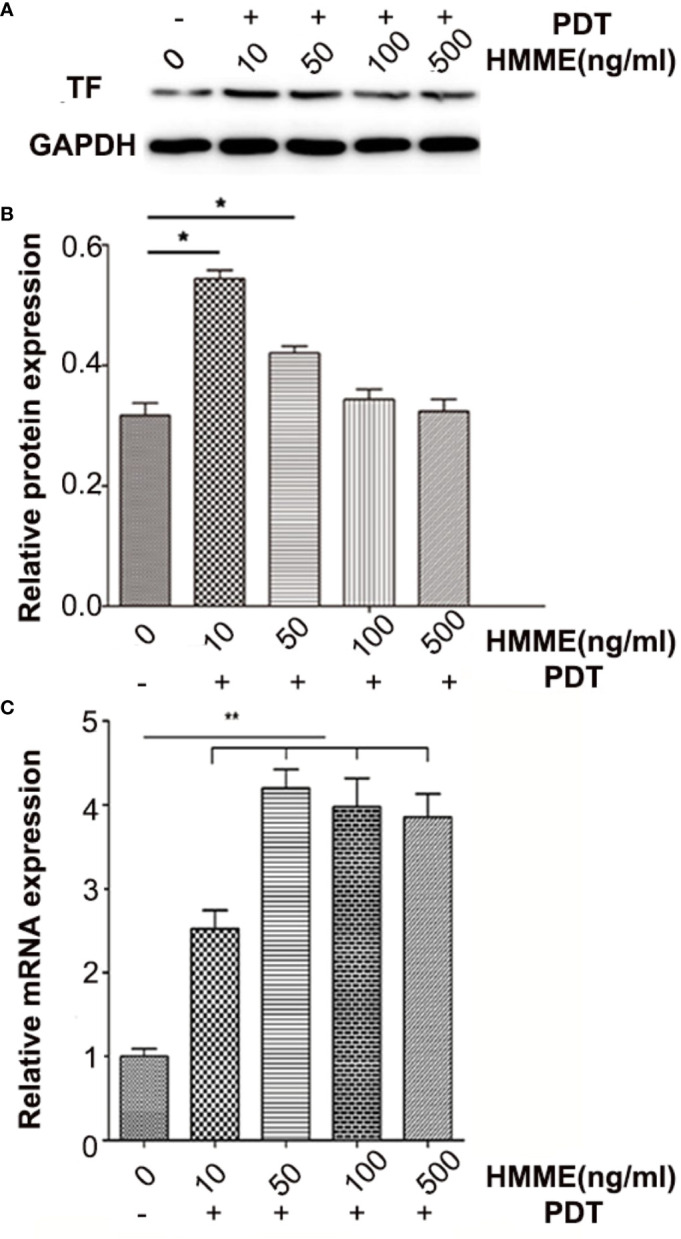
TF expression of HUVECs post-PDT. **(A)** TF protein expression was analyzed by western blotting and normalized by GAPDH and blank HUVECs; **(B)** analysis of grey-level in picture A; **(C)** relative folding of TF mRNA after normalizing to GAPDH mRNA and blank HUVECs. Data are expressed as the mean ± SD (n = 3); *p < 0.05, **p < 0.01, compared with control group.

TF expression in HUVECs was studied at transcriptional and post-transcriptional levels to evaluate the ability of PDT to induce TF production. Western blotting and PCR revealed that HUVECs expressed a certain amount of TF (**Figure 3**). At 1 h post-PDT, we observed that PDT increased TF expression during transcription (**Figure 3C**). Western blotting showed that when co-incubated with 10 or 50 ng/mL of CENPs and then given a cold light source, the expression of TF increased significantly, which was consistent with the real-time PCR results. However, there was no significant change in TF expression with CENP-HMME concentrations of 100 and 500 ng/mL (**Figure 3A** vs **B**), possibly because the excessive ROS production during PDT damaged HUVECs. These findings demonstrate that combining CENPs with PDT could increase TF expression in HUVECs. Furthermore, more ROS leads to higher TF expression within limits that can recruit more nanoparticles.

### *In Vitro* Evaluation of O_2_ Production Induced by Catalase-Encapsulated Nanoparticles in CA46 Cells

**Figure 4:**
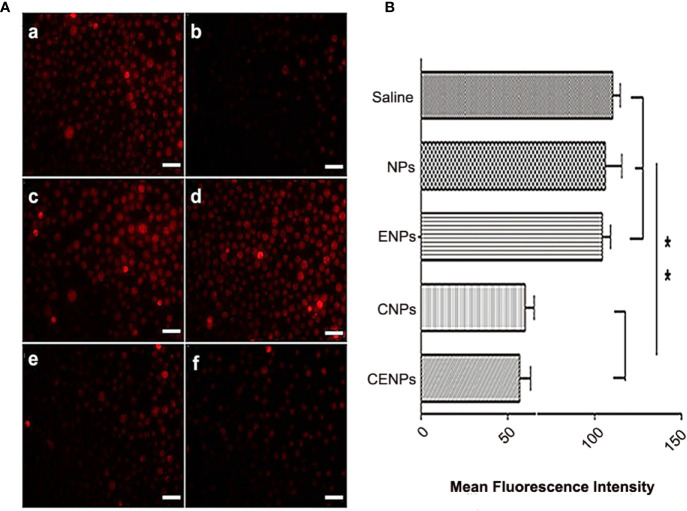
Enhanced H_2_O_2_ decomposition and O_2_ generation in CA46 cells after incubation of nanoparticles. **(A)** Level of intracellular ROS after incubation of nanoparticles. a. Control; b. NAC pre-treated; c. NPs; d. ENPs; e. CNPs; f. CENPs. **(B)** Flowcytometry data of CA46 cells stained with O_2_ indicator [Ru(dpp)_3_]Cl_2_ after different treatments were analyzed. Data are expressed as the mean ± SD (n = 3); **p < 0.01. (scale bars 100 μm).

Recent reports have indicated that most cancer cells are characterized by hypoxia and an increased generation of ROS (24, 25). Whereas strong fluorescence was observed in CA46 cells, it was significantly reduced in cells pre-treated with NAC as a ROS scavenger (**Figure 4A**), confirming the presence of endogenous ROS in CA46 cells. The intracellular fluorescence intensity of CNPs/CENPs reduced significantly, while no effect was observed on NPs/ENPs (**Figure 4A**).

[Ru(dpp)_3_]Cl_2_ was used to detect O_2_ production, and flow cytometry results showed that the fluorescence intensity of cells decreased after incubation with catalase-encapsulated nanoparticles, indicating that O_2_ was produced in the CA46 cells (**Figure 4B**). These findings suggest that CENPs could catalyze the decomposition of endogenous ROS in CA46 cells to produce O_2_.

### Fluorescence Imaging of Nanoparticles in Tumor-Bearing Mice Post-PDT

**Figure 5:**
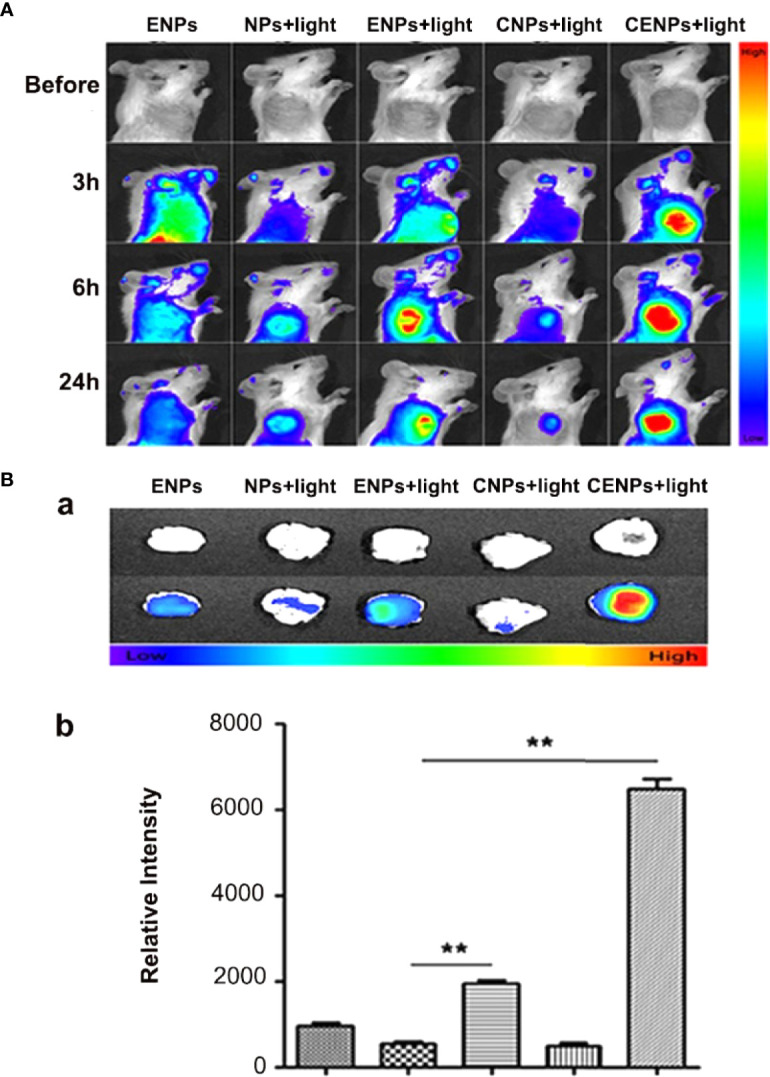
Fluorescence imaging of nanoparticles post-PDT in tumor-bearing mice. **(A)** *In vivo* multispectral fluorescent imaging of tumor-bearing mice at 3 h, 6 h, and 24 h post-PDT. **(B)** Tumors were incised at 24 h post-PDT for ex vivo multispectral fluorescent imaging. a. white and fluorescent images of tumor tissue; b. corresponding semi-quantitative fluorescence intensities of tumors. Data are expressed as the mean ± SD (n = 3); **p < 0.01.

Inspired by HUVECs targeting and catalase overcoming hypoxia in cancer cells, the *in vivo* distribution and tumor accumulation of Dir-labeled nanoparticles were studied. Compared with the ENPs group, due to PDT increasing endothelial TF expression and enhancing EPR’s impact, ENPs/CENPs accumulated further in tumors after irradiation. The NPs/CNPs plus PDT groups showed no significant accumulation because of the lack of targetability. Moreover, the increase in local nanoparticles in the CENPs plus PDT group was the most obvious and had a long retention time in the tumor tissue where the ﬂuorescence signal was maintained for 24 h. *Ex vivo* organ imaging also revealed that after PDT, the accumulation of ENPs in tumor tissues was 3.6-fold higher than that of NPs and increased to 12-fold after catalase encapsulation (**Figure 5B**).

### Biodistribution of Nanoparticles and TF Expression in Tumor Vascular Endothelium in Tumor-Bearing Mice Post-PDT

**Figure 6:**
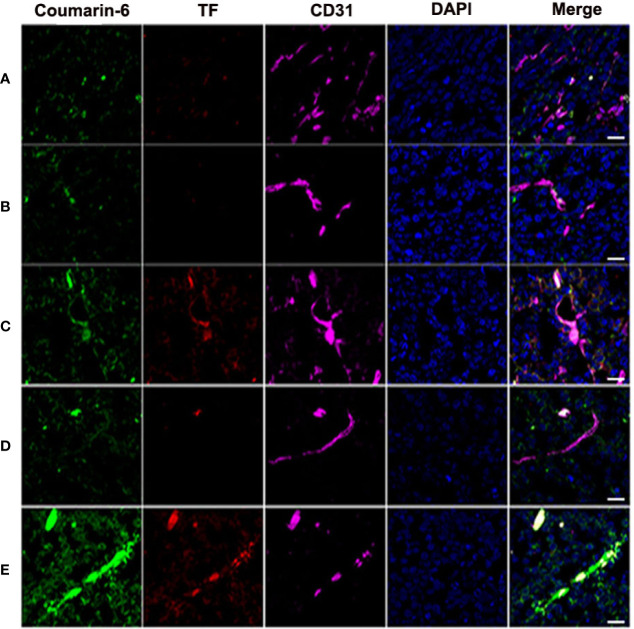
Tumors were harvested for inspection at 24 h post-PDT to estimate nanoparticle accumulation and TF expression in tumor vessels. **(A)** Coumarin-6-ENPs; **(B)** Coumarin-6-NPs plus PDT; **(C)** Coumarin-6-ENPs plus PDT; **(D)** Coumarin-6-CNPs plus PDT; **(E)** Coumarin-6-CENPs plus PDT. Frozen sections of tumors were stained with rabbit anti-rat TF polyclonal antibody and mouse-derived CD31 polyclonal antibody examined by confocal microscopy. Blue: cell nuclei. Green: coumarin-6-labelled nanoparticles. Red: TF expression. Purple: CD31. (scale bars 20 μm).

Frozen sections showed that nanoparticles were significantly abundant in blood vessels, and the expression of vascular endothelial TF increased considerably in the CENPs plus PDT group (**Figure 6**). In addition, **Figure 6E** reveals a partial CENPs aggregation outside of the vessel. These results demonstrate that CENPs could target tumor vessels and that PDT could promote their accumulation in local tumor tissues further. Compared with ENPs plus PDT, CENPs may generate O_2_ to overcome tumor hypoxia and maintain the PDT process to generate more ROS, thereby causing the tumor endothelium to continuously express more TF and ultimately recruit more “O_2_-evolving” nanoparticles.

### *In Vivo* Evaluation of O_2_ Production

**Figure 7:**
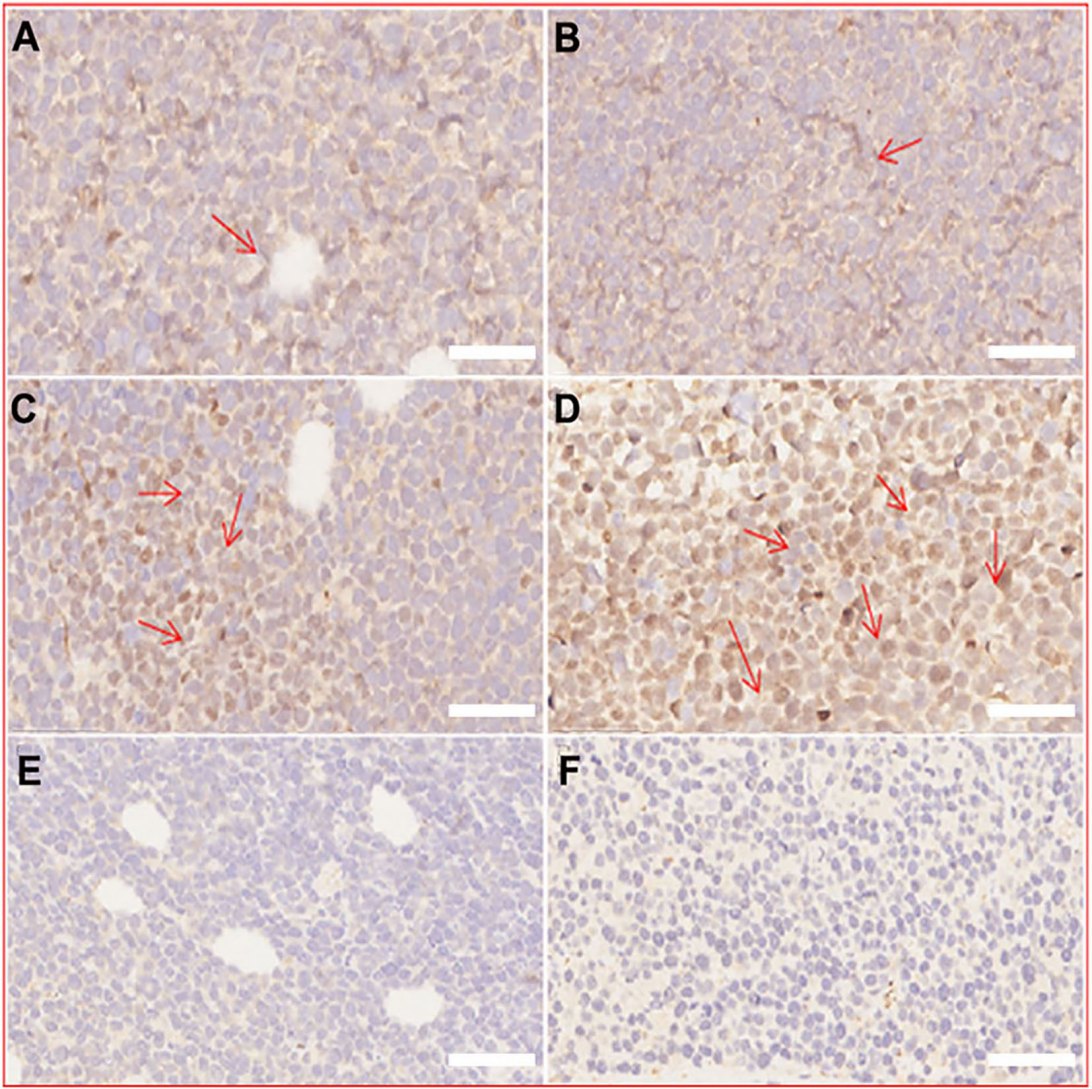
Immunohistochemical staining of HIF-α. **(A)** normal saline, **(B)** ENPs, **(C)** NPs plus PDT, **(D)** ENPs plus PDT, **(E)** CNPs plus PDT, **(F)** CENPs plus PDT. (scale bars 50 μm).

In addition to the excellent tumor vascular targeting, the efficiency of CENPs in reversing tumor hypoxia was examined using the hypoxia-inducible factor (HIF)-1α staining assay (26). HIF-1α expression is a characteristic indicator for assessing hypoxia in tumor tissues. As shown in **Figure 7**, NPs/ENPs plus PDT-treated tumor tissues displayed enhanced HIF-1α expression, compared with the non-PDT-treated group, indicating that PDT aggravated intracellular hypoxia. Decreased HIF-α levels were observed after CNPs/CENPs plus PDT treatment, primarily because of the capacity of encapsulated catalase to break down H_2_O_2_ into O_2_.

### Local Tumor Thrombosis Post-PDT

PDT-targeting blood vessels use cytotoxic ROS to destroy blood vessels and induce TF to produce microthrombi, leading to the elimination of a tumor (27). Microvascular thrombosis was observed, and tumor cell necrosis was detected around the microthrombus in the form of nuclear pyknosis and nuclear lysis (**Figure S3**).

### Evaluation of Pharmacodynamics Post-PDT

**Figure 8:**
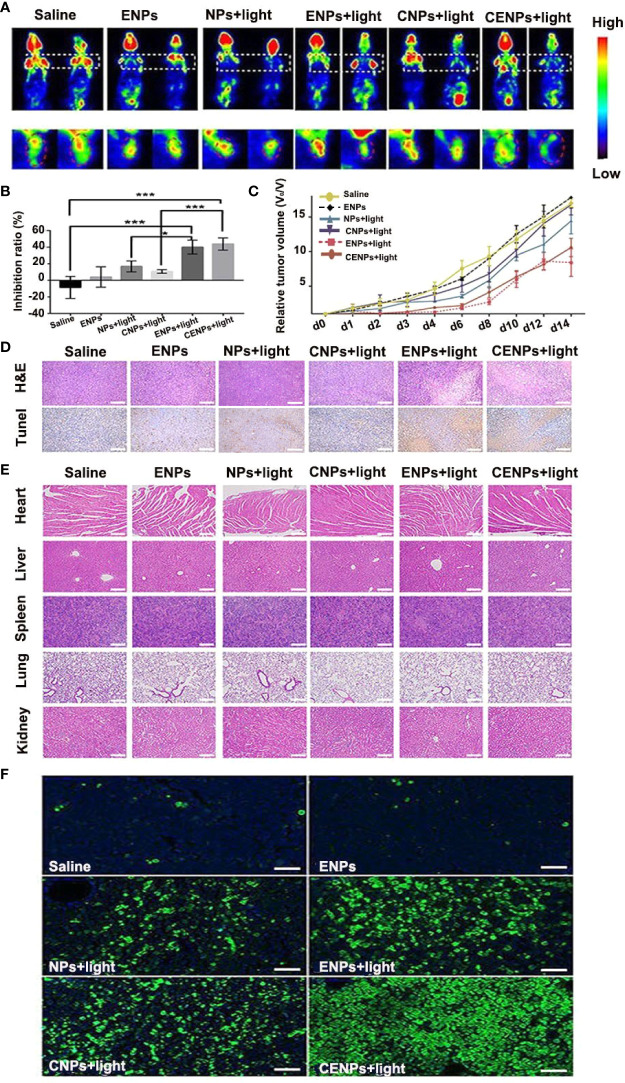
The antitumor effect and safety evaluation *in vivo*. Sections **(A–E)** correspond to the Raji mouse model. **(A)** PET imaging performed 24 h before and 24 h after PDT treatment. **(B)** Quantitative analysis of tumor growth inhibition rate by mean radioactivity of 18F-FDG. *p < 0.05, ***p < 0.001. **(C)** Relative tumor volume variation of mice treated under different conditions. **(D)** H&E staining and TUNEL staining of tumors at 24 h after PDT treatment. Apoptotic cells were identified by TUNEL assay (brown) (scale bar 100 μm). **(E)** H&E stained images of major organ slices (scale bar 200 μm). **(F)** Tumors at 24 h post-treatment and corresponding TUNEL staining of tumor slides in the Jeko-1 mouse model. (scale bar 50 μm).

For the Raji mouse model, as shown in **Figure 8A**, there was no significant change in glucose metabolism in the saline/ENPs group, whereas ENPs/CENPs significantly reduced metabolic activity after PDT, compared to NPs/CNPs plus laser. The mean radioactivity of 18F-FDG was used to quantitatively analyze inhibition rates, and the results showed that ENPs/CENPs plus PDT treatment inhibited tumor growth substantially (**Figure 8B**). As illustrated in **Figure 8C**, ENPs plus PDT and CENPs plus PDT groups exhibited higher inhibitory effects on tumor growth versus control mice treated with PBS (P = 0.0007 and P = 0.0004 at Day 6; P = 0.0208 and P = 0.0036 at Day 8). In comparison, mice injected with ENPs plus laser irradiation displayed more effective tumor inhibition than NPs plus PDT group (P = 0.0370 at Day 6). We found no significant difference between CENPs and ENPs in laser-irradiated mice (P = 0.9987 at Day 6). Next, we performed histological analysis using H&E staining and TUNEL staining and observed significant tumor necrosis with severe structural damage and more apoptotic cells in the tumors that received ENPs/CENPs plus PDT (**Figure 8D**).

The histological section images of the heart, liver, spleen, lung, and kidney tissues of mice after different treatments are presented in **Figure 8E**. Compared with those in the saline group, no obvious abnormity was found in the other groups, indicating that injecting nanoparticles with or without PDT did not have any apparent toxic effects on the major organs.

For the Jeko-1 mouse model, as displayed in **Figure 8F**, the PBS/ENPs groups showed the weakest green apoptosis signal in the TUNEL analysis, demonstrating little inhibition of tumor cell growth. A relatively strong green apoptosis signal was observed in the ENPs/CENPs plus PDT group.

## Discussion

Studies on vPDT treatment of lymphoma are rare. This research presents the first approach to treat aggressive NHL using vPDT, although the hypoxic microenvironment within a tumor limits the efficacy of oxygen-dependent PDT (26). Our investigation was performed successfully with the designed TF-cascade-targeted “O_2_-evolving” nanoparticles-CENPs.

TF is a unique biomarker for pathological neovascular endothelial cells associated with solid tumors (11). Here, the EGFP-EGF1 fusion protein, which is derived from FVII and retains its specific TF binding capability without procoagulant activity, was conjugated to PEG-PLGA nanoparticles to deliver HMME to tumor blood vessels. As shown in **Figure 2**, CENPs/ENPs had a higher HUVECs absorption efficiency after EGFP-EGF1 and TF were combined. The results in **Figure 5** also show that EGFP-EGF1-modified nanoparticles remarkably accumulated in locally abnormal TF expression tumors. The later sections revealed a similarly higher accumulation of CENPs in tumor vascular (**Figure 6**).

ROS generated from photosensitizers illuminated by light can cause vascular endothelial damage, inducing abundantly TF expression on the endothelium of tumor vasculatures. vPDT can facilitate the enhanced permeability and retention (EPR) effect of tumors; part of nanoparticles may penetrate the interior of the tumor (28). In this strategy, PDT is a trigger for the TF cascade to recruit more CENPs into a tumor. Our *in vitro* model confirmed ROS production after PDT (**Figures S2**), which promoted the TF expression of HUVECs further (**Figure 3**). In our *in vivo* evaluations, 6 h after irradiation, CENPs accumulated significantly (**Figure 5**), and the increased distribution pattern remained constant for the entirety of the assessment (24 h after PDT). **Figure 6** displays the same quantitative tendency in tumors and confirms that some CENPs leaked out of the blood vessels. All these results suggest that combining PDT with TF-targeted nanoparticles may prompt extensive recruitment of nanoparticles rather than TF-targeted nanoparticles alone.

PDT’s impact requires O_2_ as the supplement substrate *in vivo*, which is often inhibited due to the local low pO_2_ in tumor environments. Catalase-modified nanoparticles, CENPs, react with H_2_O_2_ to improve the overall O_2_ level at the site of action and, thus, fuel ROS generation in PDT. The impact of catalase on tumors was evaluated both *in vitro* and *in vivo*. Flow cytometry provided evidence that CENPs catalyzed endogenous peroxide (**Figure 4**). HIF-1α staining assay demonstrated that CENPs improved hypoxia inside tumors effectively (**Figure 7**). According to **Figure 5B**, the accumulation of CENPs in tumors was around 12-fold higher than that of NPs and 3.3-fold higher than ENPs 6 h after PDT, suggesting that catalase can enhance the production of more ROS to destroy endothelial cells and promote the aggregation of nanoparticles. Encouraged by these results, CENPs appeared to be a great potential candidate nanoplatform for photodynamic therapy of malignant lymphoma. The *in vivo* phototherapy effect was next tested in the xenograft mouse model. Notably, obvious tumor suppression was observed in mice treated with ENPs/CENPs plus PDT (**Figure 8**). Unfortunately, as exhibited in **Figure 8C**, the PDT performance was not good after Day 6. The reasons may be as follows: (1) Partial tumor necrosis induced by low-light dose fail to inhibit tumor growth (29). Modulating the therapeutic schedule, such as increasing the PDT dose, maybe potentiated PDT effectiveness. (2) Peripheral tumor blood vessels are less sensitive to PDT and that incomplete vessel shutdown leads to tumor recurrence (29, 30). Combined with other tumor therapies such as PI3K pathway inhibitors could kill tumor cells that may survive after PDT treatment (31). Moreover, “O_2_ evolving” CENPs plus PDT did not meet our expectation in terms of their tumor-killing effect strength, compared to ENPs, possibly due to (1) abnormal morphological and hemodynamic features of tumor microcirculation resulting in poor perfusion in some areas, which may have affected the delivery and uniform distribution of targeted drugs and weakened the therapeutic effect (32), and (2) the possibility of single-dose causing incomplete vascular obstruction and, thus, failing to achieve the desired killing effect (33).

Finally, the long-term curative features and the safety of CENPs combined with PDT remain major concerns. A study in a preclinical mouse model of prostate cancer demonstrated that vPDT increased the influx of immunosuppressive myeloid cells in tumors, which would suppress adaptive immune responses against remaining surviving tumors cells (34). Based on the finding, vPDT might be utilized in combination with immunotherapy (e.g., anti-CSF1R treatment) for triggering a long-lasting treatment response. Although CENPs plus PDT did not induce obvious acute side effects in animal models in healthy tissues, it could, in the long run, cause some adverse effects at varying degrees. Future investigations are, therefore, needed to assess long-term toxicity and to optimize modulation to gain maximum therapeutic benefits.

In summary, we developed tissue factor-cascade-targeted “O_2_-evolving” nanoparticles and combined them with PDT to treat lymphoma. Thanks to the EGFP-EFG1 conjugation, nanoparticles located tumor blood vessels with precision. Supposedly, after PDT, local ROS production damages the vascular endothelium and induces TF release to recruit more CENPs, causing CENPs to exude blood vessels. In addition, the “O_2_-evolving” characteristics of CENPs improve tumor hypoxia and satisfy the O_2_-dependent PDT process, thus maximizing vascular infarction and localized cancer cell killing effect. Combining this vPDT with conventional anticancer therapies offers a promising future opportunity for cancer treatment, particularly for patients with more advanced disease.

## Data Availability Statement

All datasets generated for this study are included in the article/**Supplementary Material**.

## Ethics statement

The animal study was reviewed and approved by the ethical committee of Tongji Medical College (Tongji Medical College, Huazhong University of Science and Technology, Wuhan, China).

## Author contributions

YH and WS designed the study. ZL and YY collected the data. ZL, YY, and WS analyzed the data and drafted the manuscript. All authors contributed to the article and approved the submitted version.

## Funding

This work was supported by the National Natural Science Foundation of China (Grant numbers 81302043,81500109, 81500168), the Major International Joint Research Project of China (Grant number 31620103909), the Integrated Innovative Team for Major Human Diseases Program of Tongji Medical College, the Clinical Research Physician Program of Tongji Medical College, HUST, and the Collaborative Innovation Center of Haematology, China.

## Conflict of Interest

The authors declare that the research was conducted in the absence of any commercial or financial relationships that could be construed as a potential conflict of interest.
