# Supplementary figures and images for: Tissue Factor-Targeted “O2-Evolving” Nanoparticles for Photodynamic Therapy in Malignant Lymphoma
Source: Front Oncol. 2020 Nov 10;10:524712. doi: 10.3389/fonc.2020.524712 (PMC7683716; doi:10.3389/fonc.2020.524712)

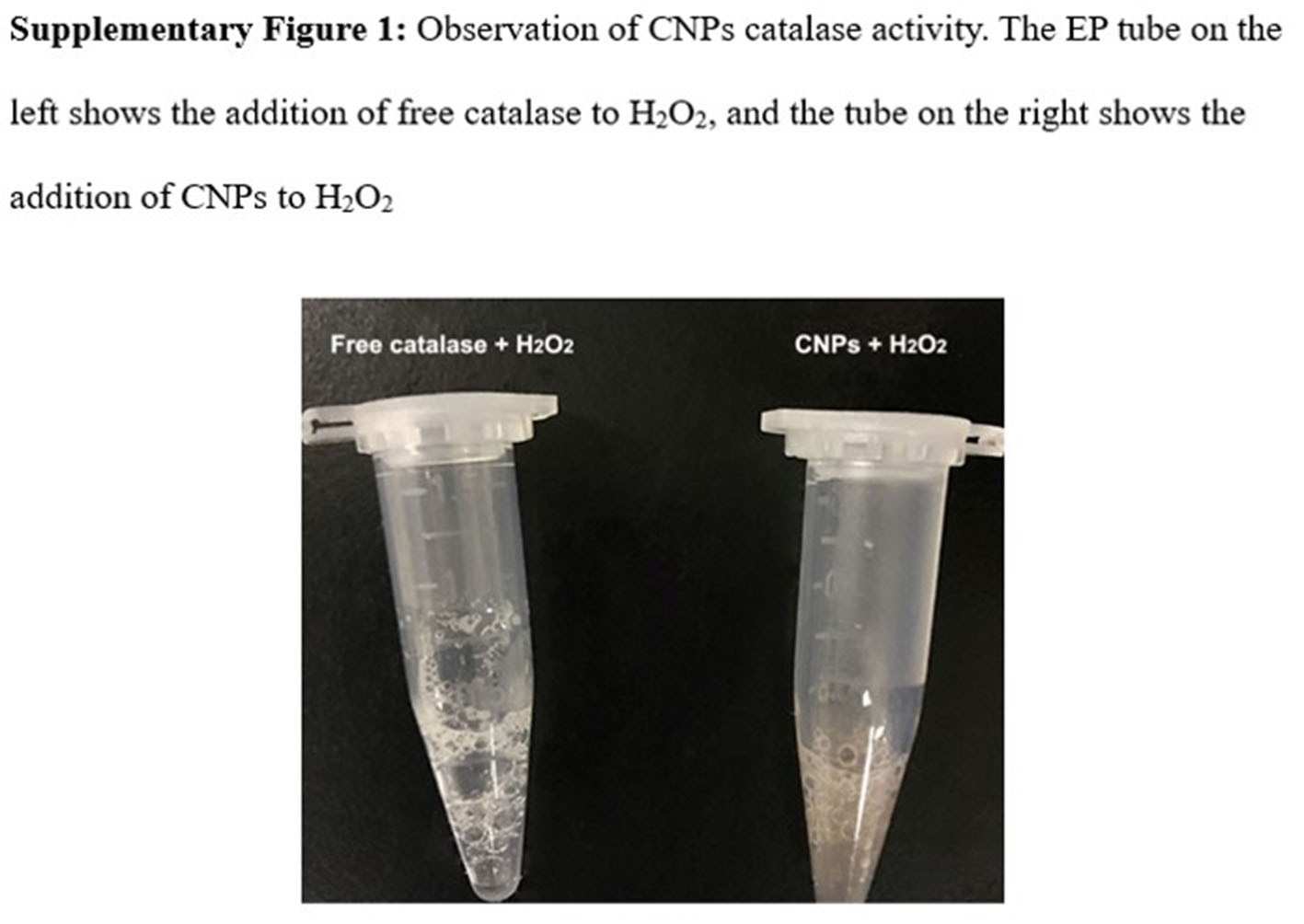

Supplement: Supplementary file 1 [file Image_1.jpg]

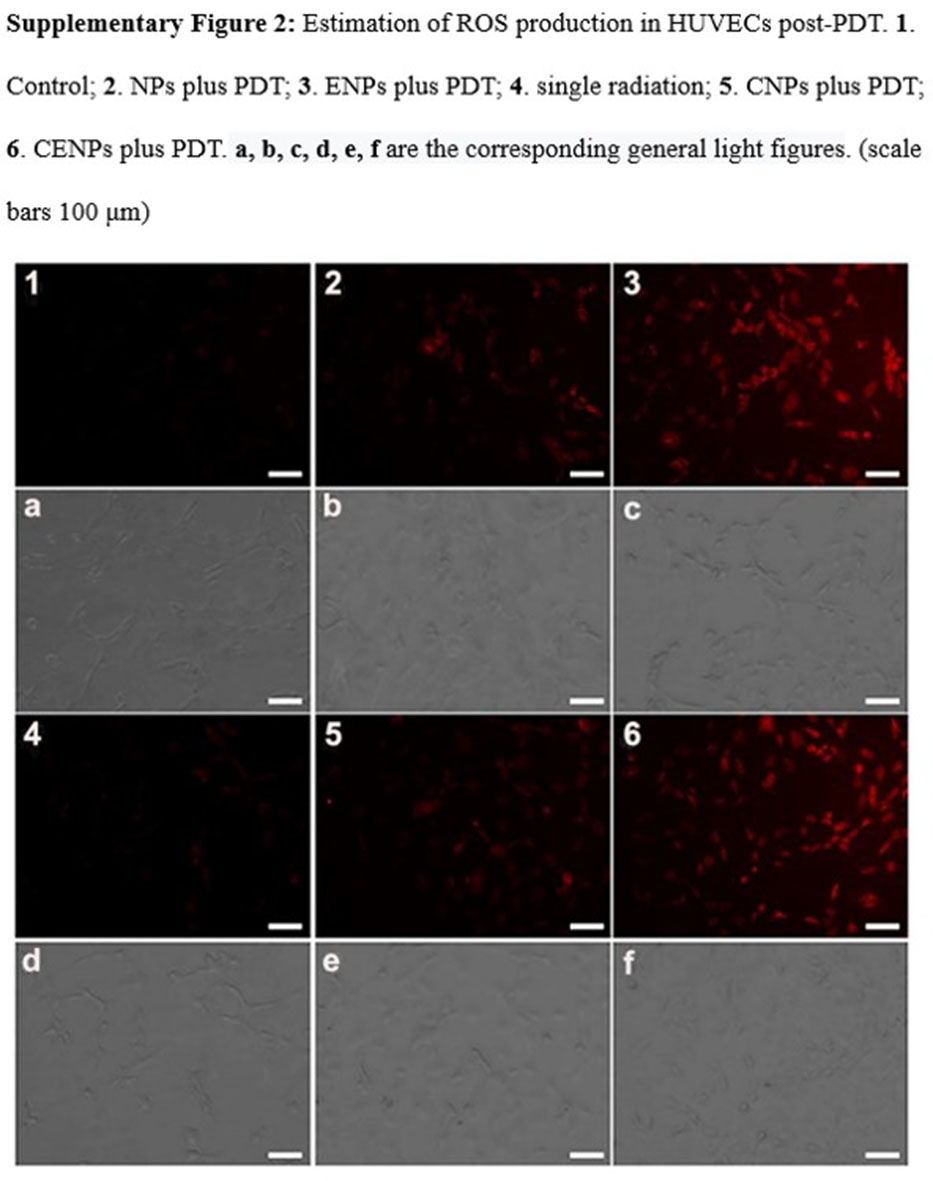

Supplement: Supplementary file 2 [file Image_2.jpg]

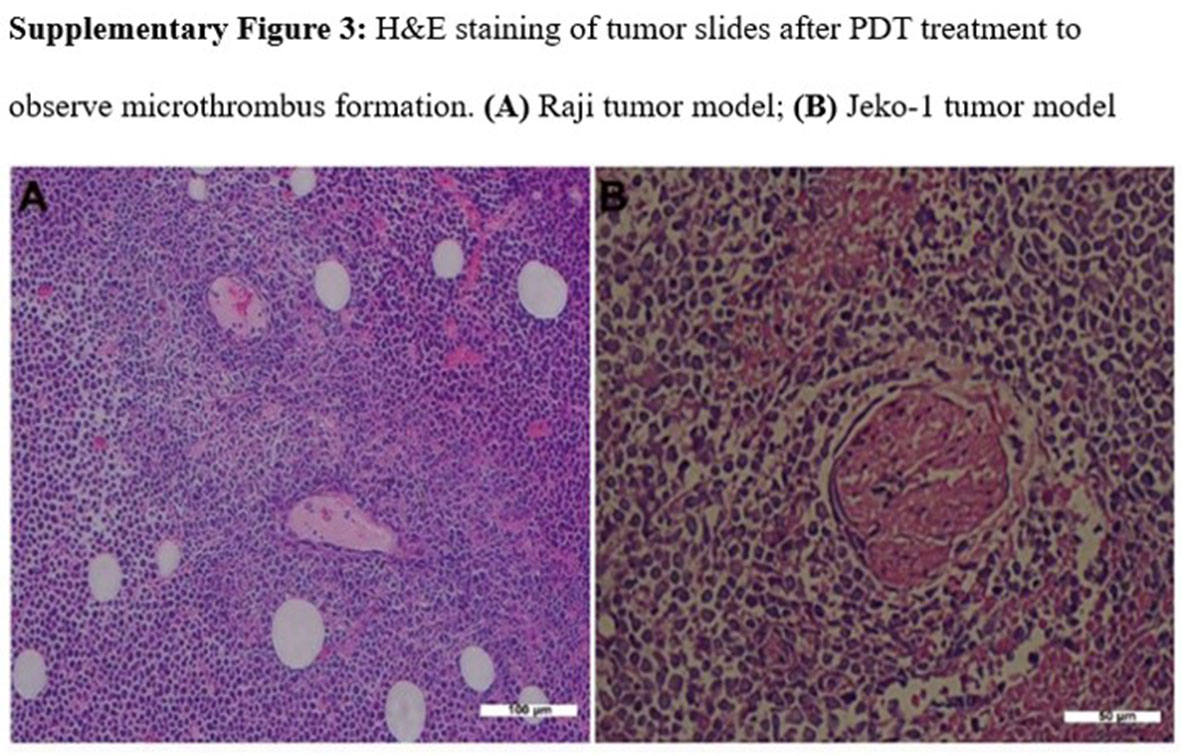

Supplement: Supplementary file 3 [file Image_3.jpg]

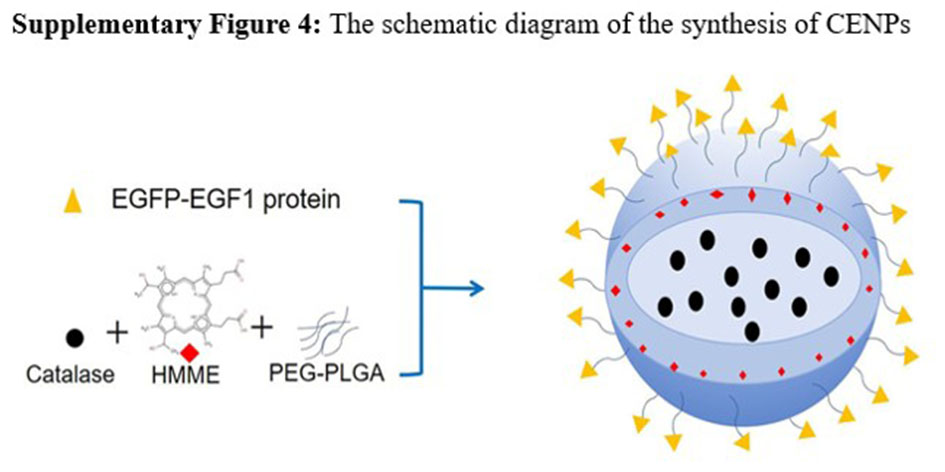

Supplement: Supplementary file 4 [file Image_4.jpg]
